# District-Level Disparities in Maternal and Child Health Performance in Manipur: A CRITIC-TOPSIS Assessment

**DOI:** 10.3390/healthcare14193143

**Published:** 2026-09-22

**Authors:** Gaurav Chandrashekhar Hajare, A. Venugopal Shanbhogue, Ujjwal, Vishwajeet Singh

**Affiliations:** 1 Directorate of Online Education, Manipal Academy of Higher Education, Manipal 576104, India; hajare.gaurav@manipal.edu (G.C.H.); venugopal.s@manipal.edu (A.V.S.); 2 Manipal Institute of Technology, Manipal Academy of Higher Education, Manipal 576104, India; ujjwal.yadav@manipal.edu

**Keywords:** maternal and child health performance, CRITIC–TOPSIS, K-means clustering, NFHS-5, regional disparity, composite index, Manipur

## Abstract

**Highlights:**

**What is already known on this topic?**

- Maternal and child health outcomes vary considerably across districts, but state-level averages can mask important local disparities.
- Composite assessment can integrate multiple healthcare and nutritional indicators to provide a broader picture of relative performance.

**What does this study add?**

- A CRITIC–TOPSIS framework integrating 11 indicators was used to assess maternal and child health performance across nine districts of Manipur.
- Imphal West showed the highest and Ukhrul the lowest composite performance, with exploratory clustering revealing distinct relative performance groupings.

**What are the implications of this study?**

- District-level assessment can identify disparities that may be obscured by state-level summaries.
- The findings support indicator-specific, differentiated district-level planning rather than reliance on composite rankings alone.

**Abstract:**

**Objectives**: This study assesses district-level disparities in maternal and child health performance across nine districts of Manipur using the CRITIC–TOPSIS approach. It aims to provide a multidimensional assessment of relative district performance and identify patterns of inter-district disparities to support differentiated maternal and child health planning. **Methods**: Eleven indicators relating to antenatal care, institutional delivery, postnatal care, immunization, breastfeeding, and nutritional outcomes of children were analyzed using data from the National Family Health Survey-5 (NFHS-5), 2019–2021. The CRITIC method was used to obtain objective weights by considering indicator variability and intercriteria correlations, and TOPSIS was used to construct the composite index and rank the districts according to their relative proximity to an ideal health performance profile. K-means clustering of the standardized composite index was used to explore relative district groupings, with alternative cluster solutions, Ward.D2 hierarchical clustering, and random-initialization stability used to assess the clustering results. **Results**: The results revealed substantial inter-district variation, with Imphal West having the highest composite index (0.8674), followed by Bishnupur (0.5851) and Imphal East (0.5564), while the lowest value was observed for Ukhrul (0.3499). Strong positive associations were observed among several components of the maternal and newborn continuum of care, particularly institutional delivery and maternal and newborn postnatal care. K-means clustering produced a three-cluster grouping comprising Imphal West in the Higher-Performance cluster; Bishnupur, Imphal East, Churachandpur, Thoubal, and Tamenglong in the Intermediate-Performance cluster; and Senapati, Chandel, and Ukhrul in the Lower-Performance cluster. Although K = 2 yielded the highest silhouette width, it primarily separated Imphal West from all remaining districts. Therefore, K = 3 was retained as an exploratory grouping because it provided greater differentiation among the remaining districts while maintaining positive silhouette values and showing complete agreement with Ward.D2 hierarchical clustering and stability across random initializations. **Conclusions**: The findings reveal a more nuanced pattern of maternal and child health performance than a simple geographical dichotomy and highlight the value of district-specific assessment. The CRITIC–TOPSIS framework, complemented by cluster analysis, provides a reproducible approach for identifying relative disparities and supporting differentiated maternal and child health planning.

## 1. Introduction

Maternal and child health is a key indicator of population health and an essential component of sustainable development. Despite considerable progress in reducing preventable maternal and child mortality, inequalities in access to and utilization of quality healthcare remain an important public health concern, particularly in low- and middle-income countries [1,2,3,4]. Improving maternal and child health is therefore a global development priority reflected in the United Nations Sustainable Development Goals (SDGs), particularly Targets 3.1 and 3.2, which focus on reducing maternal mortality and preventing avoidable deaths among newborns and children under five years of age [2,3].

Recent public health discourse has increasingly emphasized effective coverage and continuity of care rather than service availability alone. Maternal and child health performance encompasses interconnected dimensions across pregnancy, childbirth, the postnatal period, infancy, and early childhood, including maternal healthcare utilization, newborn care, immunization, breastfeeding, and nutritional outcomes [5,6]. Broader socioeconomic and contextual factors, including women’s education and empowerment, household conditions, and access to healthcare services, can also influence maternal and child health outcomes [7,8,9,10,11,12]. These factors provide important context for understanding geographical disparities but are not directly incorporated into the composite index developed in the present study.

India has made substantial progress in maternal and child health through investments in reproductive, maternal, newborn, child, and adolescent health (RMNCH+A) programmes, institutional healthcare services, and primary healthcare systems. The continuum-of-care approach is central to these initiatives, promoting sustained healthcare from pregnancy through childbirth, the postnatal period, infancy, and early childhood. Evidence from India has associated continuity across maternal and child healthcare services with greater healthcare utilization and improved child health outcomes [13,14].

Nevertheless, substantial geographical disparities persist. District-level evidence has demonstrated spatial variation in child growth failure and its association with household and socioeconomic conditions [15]. NFHS-5 similarly documents considerable geographical variation in antenatal care, institutional delivery, postnatal care, childhood immunization, and nutritional outcomes, emphasizing the importance of geographically disaggregated assessment [16]. The National Institution for Transforming India (NITI Aayog), the Government of India’s apex public-policy think tank, has also developed the Healthy States, Progressive India initiative to compare health outcomes and health-system performance across states and support evidence-informed policymaking [17]. However, state-level assessments may obscure substantial within-state variation because districts differ in healthcare access, infrastructure, demographic characteristics, and socioeconomic conditions [18].

In Manipur, a small and geographically diverse state in northeastern India, mountainous terrain, dispersed population settlements, ethnic diversity, and unequal access to healthcare resources make district-level assessment particularly relevant. Although progress has been reported toward health-related Sustainable Development Goals, disparities in maternal and child healthcare persist across geographical areas [19]. More broadly, studies across India have documented substantial regional and district-level disparities in healthcare provision, infrastructure, socioeconomic development, and population welfare [20,21,22,23,24,25,26,27,28,29]. These findings reinforce the importance of geographically disaggregated assessment for identifying local disparities that may be obscured by state-level averages.

Multidimensional approaches provide a useful framework for assessing such disparities because multiple health indicators can be evaluated jointly rather than independently. Composite indicators facilitate the integration of several dimensions into a unified framework for geographical comparison [30,31]. The Criteria Importance Through Intercriteria Correlation (CRITIC) method provides objective indicator weights based on variability and inter-criterion relationships [32], while the Technique for Order Preference by Similarity to Ideal Solution (TOPSIS) ranks alternatives according to their relative proximity to an ideal multidimensional profile [33]. Multi-criteria approaches have consequently been applied across regional planning, sustainability, healthcare-related assessment, environmental management, and socioeconomic evaluation [34,35,36,37,38,39].

Despite these developments, existing studies of maternal and child health in India have generally focused on individual outcomes, specific programmes, or broader state-level comparisons [13,14,15,16,17,18,19]. Such approaches provide valuable evidence but may not adequately reveal how districts differ when maternal healthcare utilization, postnatal care, child immunization, breastfeeding practices, and nutritional outcomes are considered jointly. This limitation is particularly relevant in Manipur, where an integrated multidimensional assessment of district-level maternal and child health performance remains limited.

Against this background, the research problem addressed in the present study is the absence of an integrated district-level assessment that simultaneously captures multiple dimensions of maternal and child health performance in Manipur. Therefore, the purpose of this study is to develop a multidimensional district-level assessment of maternal and child health performance in Manipur using NFHS-5 (2019–2021) data. Specifically, the study applies CRITIC to derive objective indicator weights, TOPSIS to construct a Composite Maternal and Child Health Performance Index and rank districts according to their relative performance, and K-means clustering to identify exploratory groupings of districts with similar levels of composite performance.

Building on the existing literature, this study makes three main contributions. First, it develops a multidimensional district-level assessment of maternal and child health performance by integrating multiple indicators through objective weighting and composite evaluation. Second, it provides localized evidence on relative disparities across the districts of Manipur, complementing assessments based on individual indicators or broader state-level patterns. Third, it integrates CRITIC–TOPSIS with robustness analyses and exploratory clustering to provide a transparent and reproducible framework for district-level assessment that may be adapted to comparable settings.

## 2. Materials and Methods

### 2.1. Maternal and Child Health Performance Indicators

The present study assessed district-level maternal and child health performance across nine districts of Manipur using data from the National Family Health Survey-5 (NFHS-5), 2019–2021. The analysis included Bishnupur, Chandel, Churachandpur, Imphal East, Imphal West, Senapati, Tamenglong, Thoubal, and Ukhrul, with the district serving as the unit of analysis (*n* = 9). The study was restricted to these districts because comparable NFHS-5 data were consistently available across all nine districts for the eleven selected indicators. Accordingly, the sample size reflects the geographical units for which comparable district-level data were available rather than a conventional individual-level sample-size determination.

The eleven indicators were selected based on their public health relevance and consistent availability and comparability at the district-level. Collectively, they represent complementary dimensions of the maternal and child health continuum, including antenatal care, institutional delivery, maternal and newborn postnatal care, childhood immunization, breastfeeding practices, and child nutritional outcomes. Other potentially relevant indicators, including maternal mortality, infant mortality, neonatal mortality, chlorhexidine cord treatment, and severe anemia among pregnant women, were not included because corresponding variables were not consistently available at the required district level in the dataset used for the analysis, rather than because of a lack of substantive importance. The selected indicator set should therefore be interpreted as a data-constrained but multidimensional representation of maternal and child health performance, rather than an exhaustive assessment of all relevant maternal and child health outcomes. The selected indicators were:

#### Maternal and Child Health Indicators

1. Mothers who had at least four antenatal care (ANC) visits during pregnancy.
2. Mothers who registered for antenatal care in the first trimester of pregnancy.
3. Births that were delivered in a health facility (Institutional delivery).
4. Mothers who received postnatal care (PNC) from a health provider within two days of delivery.
5. Newborns who received postnatal care (PNC) from a health provider within two days of birth.
6. Children aged 12–23 months who received basic vaccination.
7. Children who were breastfed within one hour of birth (Early initiation of breastfeeding).
8. Children under six months of age who are exclusively breastfed.
9. Children under five years of age who are stunted (height-for-age below -2 SD of the WHO Child Growth Standards median).
10. Children under five years of age who are wasted (weight-for-height below -2 SD of the WHO Child Growth Standards median).
11. Children under five years of age who are underweight (weight-for-age below -2 SD of the WHO Child Growth Standards median).

Conceptually, the selected indicators represent complementary stages of the maternal and child health continuum, covering antenatal care, delivery care, maternal and newborn postnatal care, childhood immunization, infant feeding practices, and child nutritional status. Their inclusion therefore reflects both their public-health relevance to maternal and child health performance and their consistent availability at the district-level in NFHS-5. Taken together, these indicators provide a multidimensional representation of service utilization, preventive child healthcare, feeding practices, and nutritional outcomes rather than relying on any single dimension of maternal and child health.

Indicator direction was specified a priori according to whether a higher observed value represented a more favorable maternal and child health condition. The first eight indicators ANC 4+ visits, first-trimester ANC, institutional delivery, maternal PNC, newborn PNC, basic vaccination, breastfeeding within one hour, and exclusive breastfeedingwere treated as benefit criteria (higher-is-better). Stunting, wasting, and underweight were treated as cost criteria (lower-is-better) because lower prevalence represents a more favorable nutritional outcome. This directional classification was subsequently incorporated into the determination of the positive and negative ideal solutions in the TOPSIS procedure.

Prior to analysis, the district-level values of all 11 indicators were verified and assembled into a complete decision matrix. The dataset contained no missing observations; therefore, no imputation was performed. The indicator columns were also checked for exact duplication to ensure that conceptually distinct indicators did not contain inadvertently repeated district-level series. No duplicate indicator columns were identified in the verified dataset. The complete district-level source data used in the analysis are reported in the Supplementary Material to facilitate independent verification and reproducibility.

### 2.2. Methodology

#### 2.2.1. Estimation of the Composite Maternal and Child Health Performance Index

A hybrid multi-criteria decision-making (MCDM) approach that combines the Criteria Importance Through Intercriteria Correlation (CRITIC) method and the Technique for Order Preference by Similarity to Ideal Solution (TOPSIS) was used to develop a district-level Composite Maternal and Child Health Performance Index. The objective indicator weights were calculated using CRITIC, which accounted for the degree of conflict between the indicators, as well as their variability. The resulting weights were subsequently incorporated into TOPSIS to evaluate each district according to its relative proximity to an ideal maternal and child health performance profile.

Let $X=\left[x_{ij}\right]$ be the raw data matrix with n=9 districts (alternatives) and m=11 maternal and child health performance indicators (criteria), where $x_{ij}$ represents the observed value of indicator j for district i. The analytical procedure was implemented as follows.

**Step 1: Normalization of the decision matrix for CRITIC Weighting**

To place the indicators on a comparable scale while accounting for their direction of preference, direction-adjusted min–max normalization was applied prior to calculating the CRITIC weights. For benefit criterion j, the normalized value was obtained as:

$${Z_{ij}}^{C}=\frac{x_{ij}-min(x_{j})}{\max\left(x_{j}\right)-min(x_{j})}$$

whereas for a cost criterion j, reverse min-max normalization was applied:

$${Z_{ij}}^{C}=\frac{{\max\left(x_{j}\right)-x}_{ij}}{\max\left(x_{j}\right)-min(x_{j})}$$

where $min(x_{j})$ and $max(x_{j})$ denote the minimum and maximum observed values of criterion j, respectively.

This transformation ensured that, in the CRITIC-normalized matrix $Z^{C}=[z_{ij}^{C}]$, higher normalized values consistently represented more favorable maternal and child health performance across all eleven criteria.

**Step 2: Estimation of objective weights using CRITIC**

The CRITIC method is applied to compute the weights $w_{j}$ for each indicator j using the following procedure:

(a) Compute the standard deviation $\sigma_{j}$ for each criterion j, reflecting its contrast intensity.
(b) Construct the correlation matrix $R=\left[r_{jk}\right]$ between all pairs of criteria, where k=1,2,3,…, m denotes the index of criteria.
(c) The total conflict associated with criterion j, was calculated as:
  $$G_{j}=\sum_{k=1}^{m}\left(1-r_{jk}\right)$$
(d) Calculate the amount of information contained in each criterion as:
  $$C_{j}=\sigma_{j}\cdot G_{j}=\sigma_{j}\cdot \sum_{k=1}^{m}\left(1-r_{jk}\right)$$

A criterion therefore receives greater information content when it exhibits greater variation across districts and/or greater conflict (lower redundancy) with the remaining criteria.

The objective CRITIC weight for criterion was then calculated as:

$$w_{j}=\frac{C_{j}}{\sum_{j=1}^{m}C_{j}},$$

subject to:

$$\sum_{j=1}^{m}w_{j}=1$$

**Step 3: Construction of Weighted Normalized Decision Matrix**

Following estimation of the CRITIC weights, TOPSIS was applied independently to the original decision matrix $X=\left[x_{ij}\right].$ To account for differences in measurement magnitude, vector normalization was performed as:

$$r_{ij}=\frac{x_{ij}}{\sqrt{\sum_{i=1}^{n}x_{ij}^{2}}}$$

The resulting vector-normalized TOPSIS matrix $R^{T}=[r_{ij}]$ was then combined with the CRITIC weights to obtain the weighted normalized decision matrix $V=\left[v_{ij}\right]$:

$$v_{ij}=w_{j}\cdot r_{ij}$$

Accordingly, the CRITIC and TOPSIS components employed distinct normalization procedures. Direction-adjusted min–max normalization was used to estimate CRITIC weights, whereas vector normalization of the original decision matrix was used for TOPSIS. This separation ensures that the objective weights are derived from comparable, direction-consistent criteria while TOPSIS retains its standard distance-based normalization structure.

**Step 4: Treatment of benefit and cost criteria and determination of ideal solutions**

The direction of each criterion was explicitly incorporated at this stage. Eight indicators—ANC 4+ visits, first-trimester ANC, institutional delivery, maternal PNC within two days, newborn PNC within two days, basic vaccination, breastfeeding within one hour, and exclusive breastfeeding—were treated as benefit criteria, for which higher values represent more favorable performance. Stunting, wasting, and underweight were treated as cost criteria, for which lower prevalence represents more favorable performance.

Accordingly, the positive ideal solution $A^{+}$ was defined as:

$$v_{j}^{+}=\left\{\begin{array}{c} {max}_{i}\left(v_{ij}\right),\;j\in B\\ {min}_{i}\left(v_{ij}\right),\;j\in C \end{array}\right.$$

and the negative ideal solution $A^{-}$ as:

$$v_{j}^{-}=\left\{\begin{array}{c} {min}_{i}\left(v_{ij}\right),\;j\in B\\ {max}_{i}\left(v_{ij}\right),\;j\in C \end{array}\right.$$

where B represents the set of benefit criteria and C represents the set of cost criteria.

Thus, stunting, wasting, and underweight were not treated as higher-is-better indicators. Their lower weighted normalized values defined the positive ideal, whereas their higher values defined the negative ideal. This ensured that lower prevalence of adverse nutritional outcomes contributed to a more favorable district assessment.

**Step 5: Calculation of distances from the ideal solutions**

For each district, Euclidean distances from the positive and negative ideal solutions were calculated as:

$$D_{i}^{+}=\sqrt{\sum_{j=1}^{m}{\left(v_{ij}-v_{j}^{+}\right)}^{2}},$$

and

$$D_{i}^{-}=\sqrt{\sum_{j=1}^{m}{\left(v_{ij}-v_{j}^{-}\right)}^{2}}.$$

A smaller $D_{i}^{+}$ indicates greater proximity to the ideal maternal and child health performance profile, whereas a larger $D_{i}^{-}$ indicates greater distance from the least favorable profile.

**Step 6: Calculation of the Composite Maternal and Child Health Performance Index**

The relative closeness of district to the positive ideal solution was calculated as:

$${CC}_{i}=\frac{D_{i}^{-}}{\left(D_{i}^{+}+D_{i}^{-}\right)},where\;0\leq {CC}_{i}\leq 1$$

The resulting closeness coefficient was used as the Composite Maternal and Child Health Performance Index. Values closer to 1 indicate greater relative proximity to the ideal multidimensional maternal and child health performance profile, whereas values closer to 0 indicate greater distance from that profile. Districts were ranked in descending order of ${CC}_{i}$, with a higher index value representing comparatively more favorable multidimensional performance.

#### 2.2.2. K-Means Cluster Analysis

Following construction of the Composite Maternal and Child Health Performance Index using CRITIC-TOPSIS, K-means clustering was employed as an exploratory unsupervised grouping procedure to identify districts with similar levels of overall maternal and child health performance. Clustering was performed on the single district-level composite index, rather than on the complete 11-indicator matrix. Thus, each of the nine districts entered the clustering analysis as one observation represented by its Composite Maternal and Child Health Performance Index.

K-means clustering was preferred to a priori division of districts into high, medium, and low performing groups because such predefined categories would require externally specified cut-off values for the composite index. No established clinical or policy thresholds exist for the CRITIC-TOPSIS composite scores used in this study. Imposing arbitrary cut-offs could therefore determine district membership independently of the observed structure of the data. In contrast, K-means provides a data-driven exploratory grouping by assigning districts according to their relative proximity in the composite-index space and minimizing within-cluster variation. The resulting clusters were labelled Higher-Performance, Intermediate-Performance, and Lower-Performance only after examining their relative composite-index values. These labels describe relative maternal and child health performance within the study sample and do not represent absolute clinical, socioeconomic, or developmental categories.

Accordingly, K-means was not intended to identify latent multidimensional health profiles or to establish definitive district categories. Rather, it was used to derive reproducible, data-driven strata along the composite-performance continuum without imposing arbitrary cut-points. Given the small number of districts (*n* = 9), the resulting clusters were treated as exploratory groupings, and their robustness was examined using alternative cluster solutions, silhouette diagnostics, Ward.D2 hierarchical clustering, random-initialization stability, and a multidimensional indicator-profile sensitivity analysis.

Prior to clustering, the composite index was standardized to have a mean of zero and a standard deviation of one. Because clustering was based on a single variable, this transformation does not alter the relative ordering of districts but provides a standardized scale for distance-based clustering and associated validation procedures.

For a specified number of clusters, K-means partitions the observations into mutually exclusive groups by minimizing the total within-cluster sum of squares (WSS):

{∑k=1K∑i∈Gk(zi−μk)2}G1, G2,…,Gkminimize,

where $G_{k}$ represents cluster k, $z_{i}$ is the standardized composite index for district i, and $\mu_{k}$ denotes the centroid of cluster k. The analysis was implemented using the Hartigan–Wong algorithm [40], with a maximum of 1000 iterations and 100 random starts for each candidate solution to reduce dependence on the initial centroid configuration.

Candidate solutions with K=2, K=3, and K=4 and were systematically compared. The number of clusters was evaluated using multiple complementary criteria rather than relying on a single diagnostic. These included the within-cluster sum of squares (elbow criterion), average silhouette width, gap statistic, cluster-size distribution, agreement with Ward.D2 hierarchical clustering, random-seed stability, and substantive interpretability of the resulting district groupings.

For each candidate solution, silhouette widths were calculated from Euclidean distances between the standardized composite-index values. The silhouette coefficient for district i was defined as:

$$s\left(i\right)=\frac{b\left(i\right)-a(i)}{max\{a\left(i\right),b(i)\}},$$

where a(i) represents the average dissimilarity of district i from observations in its assigned cluster and b(i) represents its lowest average dissimilarity from observations belonging to another cluster. Values approaching 1 indicate stronger cluster assignment, values around zero indicate boundary or singleton observations, and negative values suggest potentially inappropriate assignments.

The elbow criterion was evaluated from changes in total WSS across candidate values of K. The gap statistics were additionally estimated using 500 Monte Carlo bootstrap replications to provide an independent assessment of cluster structure.

Because K-means solutions can depend on initialization, a separate random-seed sensitivity analysis was conducted. For each candidate value of K, the clustering procedure was repeated across 100 different random seeds (1–100), with each run itself using 100 random starts. Agreement between each resulting partition and the reference solution was quantified using the Adjusted Rand Index (ARI), where an ARI of 1 represents identical cluster membership.

As an additional algorithmic sensitivity check, agglomerative hierarchical clustering was performed on the same standardized composite index using Euclidean distance and Ward’s minimum-variance method (Ward.D2). The resulting partitions for K=2, K=3, and K=4 were compared with the corresponding K-means partitions using the ARI.

Given the small number of districts (n=9), clustering was used as an exploratory descriptive stratification of relative district performance, rather than as inferential evidence for statistically distinct latent populations. Accordingly, the final cluster solution was evaluated by considering statistical diagnostics, stability, parsimony, cluster composition, and substantive interpretability together.

#### 2.2.3. Disparity Parameters

District-level composite index scores were analyzed to quantify the magnitude and distribution of disparities in maternal and child health performance across the nine districts of Manipur. Absolute, relative, robust, and distributional measures of dispersion and inequality were computed.

(a) **Range-Based Measures**

To establish a minimum and maximum level of performance for district-level composite index scores, the lowest and highest values were identified. The absolute range was calculated as:

$$Absolute\;Range=\max\left(CC_{i}\right)-min(CC_{i})$$

where $CC_{i}$ denotes the Composite Maternal and Child Health Performance Index for district i. A larger range indicates greater separation between the highest- and lowest-performing districts.

(b) **Relative Dispersion**

**The coefficient of variation (CV) was calculated as**:

$$CV=\frac{s}{\bar{C}C}\times 100$$

where s  is the standard deviation and $\bar{C}C$ is the mean composite index across the nine districts. The CV expresses the standard deviation relative to the mean and facilitates assessment of the relative dispersion in district-level index values.

(c) **Robust Dispersion Measure**

The interquartile range (IQR) was calculated as:

$$IQR=Q_{3}-Q_{1}$$

where $Q_{1}$ and $Q_{1}$ represent the first and third quartiles, respectively. The IQR measures the spread of the middle 50% of district-level index values and is less sensitive to extreme observations than the absolute range.

(d) **Inequality Measures**

To complement the dispersion measures, the Gini coefficient and Theil indices were calculated to illustrate inequality in the distribution of the composite index across districts.

The **Gini coefficient** measures the relative inequality in the distribution of index values, with values closer to zero representing greater equality and larger values indicating greater inequality.

The **Theil T index** was calculated as:

$$T_{T}=\frac{1}{n}\sum_{i=1}^{n}\frac{CC_{i}}{\bar{C}C}ln(\frac{CC_{i}}{\bar{C}C})$$

**Theil L Index (mean log deviation) was calculated as:**

The Theil L index emphasizes disparities at the lower end of the distribution:

$$T_{L}=\frac{1}{n}\sum_{i=1}^{n}ln(\frac{\bar{C}C}{CC_{i}})$$

where n=9 represents the number of districts. Both Theil measures equal zero under complete equality and increase as inequality in the distribution of composite index values increases. Theil T is relatively more sensitive to differences toward the upper end of the distribution, whereas Theil L places relatively greater emphasis on differences toward the lower end.

Together, the range, CV, IQR, Gini coefficient, and Theil indices provide complementary measures of the magnitude and distribution of inter-district disparity in maternal and child health performance.

#### 2.2.4. Sensitivity Analysis of the CRITIC–TOPSIS Composite Index

To assess the robustness of the Composite Maternal and Child Health Performance Index to the weighting specification and the influence of individual district observations, two complementary sensitivity analyses were performed.

First, an equal-weight TOPSIS analysis was conducted by assigning an identical weight of $w_{j}=\frac{1}{11}$ to each of the eleven indicators. All other components of the TOPSIS procedure, including vector normalization, treatment of benefit and cost criteria, and determination of the positive and negative ideal solutions, were retained as in the baseline CRITIC–TOPSIS analysis. Agreement between the baseline CRITIC–TOPSIS ranking and the equal-weight TOPSIS ranking was evaluated using Spearman’s rank correlation coefficient and absolute changes in district ranks.

Second, a leave-one-district-out (LODO) sensitivity analysis was undertaken to examine whether the CRITIC weights and resulting district rankings were disproportionately influenced by any individual district. Each of the nine districts was sequentially excluded, and the complete CRITIC weighting and TOPSIS procedure was recalculated using the remaining eight districts. For each iteration, the resulting ranking was compared with the corresponding baseline CRITIC–TOPSIS ranking restricted to the same eight districts using Spearman’s rank correlation coefficient. Variation in the CRITIC weights across the nine omission scenarios was also examined to evaluate the stability of the data-derived weighting structure.

These sensitivity analyses were undertaken to assess the extent to which the composite scores and district rankings depended on the CRITIC weighting structure and the influence of individual district observations. The equal-weight specification provides a direct alternative to the CRITIC-derived weighting scheme, while the leave-one-district-out analysis evaluates the influence of individual districts on the resulting CRITIC weights and district rankings.

#### 2.2.5. Sensitivity Analysis of the Clustering Strategy

To assess whether the score-based district grouping was also reflected in the underlying maternal and child health profiles, an additional multidimensional clustering sensitivity analysis was performed using all eleven indicators. The three cost indicators—stunting, wasting, and underweight—were direction-adjusted so that higher values consistently represented more favorable outcomes, after which all eleven indicators were standardized using z-scores. K-means clustering was applied to the resulting standardized 11-indicator matrix for K = 2, K = 3, and K = 4, using the Hartigan–Wong algorithm with 100 random starts and a maximum of 1000 iterations.

Cluster performance was assessed using within-cluster sum of squares (WSS), average and district-level silhouette widths, cluster-size distribution, agreement with Ward.D2 hierarchical clustering using the Adjusted Rand Index (ARI), and sensitivity to random initialization across 100 seeds. For K = 3, agreement between the multidimensional indicator-profile grouping and the primary composite-index-based grouping was additionally evaluated using ARI. This analysis was intended as a sensitivity assessment of whether similarity in composite-index scores corresponded to similarity across the underlying multidimensional indicator profiles.

#### 2.2.6. Software and Computational Implementation

All statistical analyses and visualizations were performed in R (version 4.6.1) using scripts developed by the authors for the present study. The computational workflow combined author-written scripts with established functions available in standard R packages for data processing, CRITIC–TOPSIS computation, disparity measurement, K-means and hierarchical clustering, sensitivity analyses, and graphical visualization. No standalone ready-made software was used to generate analytical results. The R scripts required to reproduce the analyses are provided in Supplementary Files.

## 3. Results

### 3.1. Selection of the Indicators

Eleven indicators representing maternal healthcare utilization, postnatal care, child immunization, breastfeeding practices, and child nutritional outcomes were included in the assessment of maternal and child health performance across the nine districts of Manipur. Based on the expected direction of favorable health performance, eight indicators were classified as benefit criteria (higher-is-better), whereas stunting, wasting, and underweight were classified as cost criteria (lower-is-better). The classification and direction of the indicators are presented in Table 1.

Together, these indicators provide a multidimensional representation of maternal and child health performance across the included districts of Manipur.

### 3.2. Descriptive Statistics

Descriptive statistics were computed for the eleven maternal and child health performance indicators to examine their central tendency, dispersion, and distribution across the nine districts of Manipur. Table 2 presents the mean, standard deviation (SD), median, minimum, maximum, range, skewness, kurtosis, standard error (SE), and coefficient of variation (CV) for each indicator.

The indicators displayed appreciable differences in their levels and dispersion across districts. Among the maternal healthcare indicators, first-trimester ANC recorded the highest mean coverage (76.09%), followed by institutional delivery (71.24%) and ANC with four or more visits (70.64%). Postnatal care coverage was comparatively lower, with mean values of 64.29% for mothers and 60.99% for newborns within two days. Among child-care indicators, exclusive breastfeeding averaged 69.84%, basic vaccination 64.81%, and breastfeeding within one hour of birth 55.26%.

Relative variability differed substantially across indicators. Newborn PNC within two days showed the highest CV (29.83%), closely followed by maternal PNC (29.47%) and institutional delivery (29.00%). ANC 4+ also displayed considerable variation (CV = 25.32%). In contrast, exclusive breastfeeding showed the lowest relative dispersion (CV = 8.06%), followed by first-trimester ANC (CV = 12.74%). The ranges similarly indicate wide district-level differences in newborn PNC (55.7 percentage points), ANC 4+ (54.6 percentage points), maternal PNC (53.8 percentage points), and institutional delivery (51.1 percentage points).

For the adverse nutritional indicators, the mean prevalence of stunting, wasting, and underweight was 24.62%, 9.94%, and 13.77%, respectively. Stunting showed greater relative district-level variation (CV = 27.18%) than underweight (21.74%) and wasting (16.36%).

Most indicators exhibited relatively modest skewness, although basic vaccination showed a comparatively stronger negative skew (−1.0461). Kurtosis was negative for all indicators, although its magnitude varied considerably. Given the small number of district-level observations (n=9), skewness and kurtosis are reported primarily as descriptive characteristics rather than as formal evidence regarding distributional form.

### 3.3. Correlation Analysis Among the Indicators

Pearson correlation coefficients were calculated to descriptively examine the direction and strength of district-level linear associations among the eleven maternal and child health performance indicators. Given the nine district-level observations, the coefficients were used to characterize patterns of co-variation rather than for inferential hypothesis testing. The correlation matrix is presented in Table 3, while the corresponding heatmap provides a visual representation of the overall correlation structure.

The matrix shows strong positive associations among several indicators representing maternal and newborn healthcare utilization. The largest correlation was observed between maternal and newborn PNC within two days (r=0.974), followed by institutional delivery with maternal PNC (r=0.971) and newborn PNC (r=0.936). ANC 4+ was also strongly positively correlated with first-trimester ANC (r=0.917), institutional delivery (r=0.849), basic vaccination (r=0.855), maternal PNC (r=0.849), and newborn PNC (r=0.843). At the district level, these patterns indicate that relatively higher coverage of several maternal and newborn healthcare services tended to occur together.

Several inverse relationships were observed between healthcare-utilization indicators and adverse nutritional outcomes. The strongest negative correlation in the matrix was between first-trimester ANC and wasting (r=−0.691). Stunting was inversely correlated with maternal PNC (r=−0.657), institutional delivery (r=−0.647), and newborn PNC $\left(r=-0.605\right).$ Exclusive breastfeeding was negatively correlated with underweight (r=−0.452) and wasting (r=−0.422). Among the nutritional indicators themselves, stunting and underweight showed a moderate positive correlation (r=0.491), whereas the relationships of wasting with stunting (r=−0.079) and underweight (r=−0.082) were weak.

Breastfeeding within one hour exhibited a different correlation pattern from several healthcare-utilization indicators, including inverse correlations with first-trimester ANC (r=−0.574), institutional delivery (r=−0.482), ANC 4+ (r=−0.448), and maternal PNC (r=−0.444). These relationships should be interpreted as descriptive district-level co-variation rather than evidence that one indicator influences another. The corresponding heatmap (Figure 1) provides a visual representation of these inter-indicator relationships.

The observed correlation structure is also relevant to the subsequent CRITIC analysis because CRITIC incorporates both the variability of individual indicators and their correlation with other criteria when deriving objective weights. Thus, indicators containing relatively less redundant information can receive greater information content in the weighting procedure. Accordingly, the strong correlations observed among several maternal healthcare indicators suggest some redundancy in the information captured by these criteria, which is explicitly accounted for within the CRITIC weighting procedure.

### 3.4. Composite Maternal and Child Health Performance Index Based on CRITIC–TOPSIS

The CRITIC method was applied to derive objective weights for the eleven maternal and child health performance indicators by jointly considering the variability of each indicator and its degree of conflict with the remaining indicators. The resulting normalized standard deviations, conflict measures, information content, and CRITIC weights are presented in Table 4.

The CRITIC weights show that breastfeeding within one hour of birth received the largest weight (0.1904), followed by wasting (0.1269), exclusive breastfeeding (0.1039), stunting (0.0994), and underweight (0.0931). In contrast, ANC 4+ received the smallest weight (0.0596), followed by first-trimester ANC (0.0621) and newborn PNC within two days (0.0624).

These weights should be interpreted as measures of the information contribution of the indicators within the CRITIC framework, rather than as estimates of their clinical or causal importance. In particular, the comparatively large weight assigned to breastfeeding within one hour reflects its combination of district-level variability and relatively high conflict with the other indicators, as reflected by its conflict value of 13.4847, the largest among the eleven criteria. The weighting procedure therefore reduces the influence of comparatively redundant information while assigning greater weight to criteria that contribute relatively distinct information to the multidimensional assessment.

The CRITIC weights were subsequently incorporated into TOPSIS to determine each district’s relative proximity to the positive ideal maternal and child health performance profile. For the eight benefit criteria, higher weighted-normalized values represented the positive ideal, whereas for stunting, wasting, and underweight, lower values represented the positive ideal. The complete vector-normalized matrix, weighted matrix, and positive and negative ideal solutions are provided in Supplementary File S1 to facilitate reproducibility.

**TOPSIS-based Composite Index and District Ranking**

The resulting distances from the positive ideal $\left(D_{i}^{+}\right)$ and negative ideal $\left(D_{i}^{-}\right)$, Composite Maternal and Child Health Performance Index values, and district rankings are presented in Table 5. Higher index values indicate greater relative proximity to the ideal multidimensional profile.

The composite index reveals substantial differences in relative maternal and child health performance across the nine districts. Imphal West ranked first with an index value of 0.8674, substantially higher than the remaining districts and indicating the closest proximity to the positive ideal profile. Bishnupur ranked second (0.5851), followed by Imphal East (0.5564). Churachandpur (0.5173) and Thoubal (0.5167) recorded very similar composite scores, although Churachandpur retained a marginally higher ranking based on the unrounded values.

At the lower end of the distribution, Senapati recorded an index of 0.4291, followed by Chandel (0.3798), while Ukhrul recorded the lowest composite index (0.3499). These rankings represent relative multidimensional performance based on the eleven indicators and their CRITIC-derived weights and should not be interpreted as causal assessments of the factors responsible for district performance.

**Inter-District Disparity in the Composite Index**

To complement the district ranking, the distribution of composite index values was evaluated using measures of dispersion and inequality. The results are presented in Table 6.

The mean composite index across the nine districts was 0.5205, with a standard deviation of 0.1519. The index ranged from 0.3499 in Ukhrul to 0.8674 in Imphal West, producing an absolute range of 0.5175. The coefficient of variation of 29.17% indicates appreciable relative dispersion in composite performance across districts.

The inequality measures provide complementary information on the distribution of district scores. The Gini coefficient was 0.1411, while the Theil T and Theil L indices were 0.0350 and 0.0333, respectively. These values indicate that although district-level composite performance is not uniformly distributed, overall inequality in the distribution of index scores is relatively limited. The comparatively large distance between the minimum and maximum scores is influenced particularly by the high composite value observed for Imphal West, whereas the Gini and Theil measures consider the distribution of scores across all nine districts.

#### Sensitivity and Robustness of the CRITIC–TOPSIS Composite Index

The robustness of the composite index was evaluated under an alternative equal-weight specification and through leave-one-district-out (LODO) analysis. The results are summarized in Table 7.

Under the equal-weight specification, in which each of the eleven indicators received an identical weight of $\frac{1}{11}$, the resulting district ranking showed strong agreement with the baseline CRITIC–TOPSIS ranking (Spearman’s ρ=0.9500). The maximum absolute rank displacement was limited to one position. Imphal West retained the highest rank and Ukhrul retained the lowest rank, while changes were confined to adjacent positions among intermediate-ranked districts. Thus, the broad ordering of districts was not substantially altered when the CRITIC-derived weights were replaced by equal weights.

The LODO analysis provided further evidence of ranking stability. Across the nine district-omission scenarios, the mean Spearman rank correlation between the recalculated rankings and their corresponding baseline rankings was 0.9841, with individual correlations ranging from 0.9524 to 1.000. The maximum rank displacement observed in any iteration was again limited to one position (Table 7). These results indicate that exclusion of any single district did not materially alter the relative ordering of the remaining districts.

The stability of the CRITIC weights was also examined across the LODO iterations. Breastfeeding within one hour, which received the largest baseline CRITIC weight (0.1904), retained a comparatively high weight across the omission scenarios, with a mean weight of 0.1917 and a range of 0.1685–0.2108. This indicates that its relatively large statistical information contribution was not attributable to the inclusion of a single district. Nevertheless, the observed variation in the weights reinforces that CRITIC weights represent data-dependent statistical information contributions rather than fixed measures of clinical importance.

Overall, the sensitivity analyses demonstrate that the broad district-ranking structure remained stable under both an alternative weighting specification and the sequential exclusion of individual districts. However, the minor rank changes observed among intermediate-ranked districts indicate that small ordinal differences between closely positioned districts should be interpreted cautiously.

### 3.5. Exploratory Grouping of Districts Using K-Means Clustering

K-means clustering was applied to the standardized Composite Maternal and Child Health Performance Index to obtain an exploratory grouping of the nine districts according to their relative multidimensional performance. Candidate solutions with K=2, K=3 and K=4 were compared using within-cluster sum of squares (WSS), average silhouette width, gap statistic, cluster-size distribution, agreement with Ward.D2 hierarchical clustering, and random-seed stability. Comparative diagnostics are presented in Table 8.

The comparative diagnostics in Table 8 show that K = 2 produced the highest average silhouette width (0.6528) but resulted in a highly asymmetric 1-versus-8 partition that provided limited differentiation among the majority of districts. As shown in Figure 2, WSS declined substantially from K = 2 to K = 3, while the improvement beyond K = 3 was comparatively smaller. Thus, although the silhouette criterion favored K = 2, the K = 3 solution provided greater differentiation among the remaining districts while maintaining a positive average silhouette width.

The gap statistic (Figure 3) did not provide an unequivocal basis for selecting a single cluster solution and was therefore treated as a supplementary diagnostic rather than the primary selection criterion.

Considering the diagnostics jointly, K=3 was retained as the final exploratory grouping. Although K=2 produced the highest silhouette coefficient, it generated a coarse 1-versus-8 partition. The K=3 solution retained a satisfactory average silhouette width of 0.5602, contained no negative silhouette coefficients, substantially reduced WSS, and produced a more informative three-level stratification of district performance. Increasing the number of clusters to four reduced the average silhouette width further and divided the intermediate group into smaller subgroups.

For transparency, the silhouette results for all three candidate solutions are shown in Figure 4.

The retained three-cluster partition grouped Imphal West in the Higher-Performance cluster; Bishnupur, Imphal East, Churachandpur, Thoubal, and Tamenglong in the Intermediate-Performance cluster; and Senapati, Chandel, and Ukhrul in the Lower-Performance cluster. The cluster membership and index profiles are reported in Table 9.

The Higher-Performance cluster consisted only of Imphal West, with a composite index of 0.8674. The five-district Intermediate-Performance cluster had a mean index of 0.5317 (SD = 0.0395, CV = 7.44%) and ranged from 0.4831 to 0.5851. The three-district Lower-Performance cluster had a mean index of 0.3863 (SD = 0.0400, CV = 10.35%) and ranged from 0.3499 to 0.4291. Since the Higher-Performance cluster contained only one district, a within-cluster SD and CV were not defined for that group.

The geographic distribution of the retained clusters is presented in Figure 5 using the 2011 district administrative boundaries corresponding to the nine-district framework used in the analysis. The district boundaries are based on the Census of India 2011 Administrative Atlas of Manipur published by the Office of the Registrar General & Census Commissioner, India, Government of India [41]. Under this administrative framework, Imphal East and Imphal West are represented as separate adjoining districts. The colors in Figure 5 represent the K-means cluster groupings, while the district labels report the corresponding Composite Maternal and Child Health Performance Index values.

The clustering solution was additionally compared with Ward.D2 hierarchical clustering based on Euclidean distances of the same standardized composite index. The K=3 K-means and hierarchical partitions showed complete agreement (ARI = 1.000). The dendrogram also distinguished Imphal West at a substantially greater linkage height and reproduced the remaining lower- and intermediate-index group structure. As shown in Figure 6, Ward.D2 hierarchical clustering reproduced the three-cluster K-means partition.

Initialization sensitivity was assessed across 100 random seeds, with each K-means estimation using 100 random starts. For K=3, the mean, median, minimum, and maximum ARI were all 1.000, and 100% of the seed-specific solutions reproduced the reference partition. The same perfect seed stability was observed for K=2 and K=4. These results indicate that the observed partitions were not sensitive to the tested K-means initializations.

Finally, the original eleven indicators were used only to characterize the resulting clusters after grouping; they were not used as the input variables for K-means. The radar profile in Figure 7 shows the average normalized indicator performance within each K=3 cluster. For visualization, the three adverse nutritional indicators—stunting, wasting, and underweight—were direction-adjusted so that higher radial values consistently represent more favorable outcomes.

The radar profiles show a generally broader favorable performance profile for the Higher-Performance cluster across most service-utilization and direction-adjusted nutritional dimensions. The Intermediate-Performance cluster occupies an intermediate position on many indicators, whereas the Lower-Performance cluster records relatively lower normalized performance across several dimensions. These profiles are descriptive summaries of the index-derived groups and should not be interpreted as evidence that any individual indicator caused the observed cluster membership.

### 3.6. Multidimensional Indicator-Profile Clustering Sensitivity Analysis

To examine whether the district groupings derived from the composite index were also reflected in the underlying maternal and child health performance profiles, an additional sensitivity analysis was conducted by applying K-means clustering directly to the eleven direction-adjusted and standardized indicators. Candidate solutions with K=2, K=3, and K=4 were evaluated using within-cluster sum of squares (WSS), average and minimum silhouette widths, agreement with Ward.D2 hierarchical clustering, and stability across random initializations. The comparative results are presented in Table 10.

Among the candidate solutions, the K=2 partition produced the highest average silhouette width (0.3184), compared with 0.1651 for K=3 and 0.1407 for K=4 (Table 10). The corresponding WSS decreased from 45.3388 for K=2 to 35.5590 for K=3 and 26.8155 for K=4. Thus, although increasing the number of clusters reduced within-cluster variation, the silhouette results did not identify K=3 as the statistically strongest solution. This finding is consistent with the primary composite-index analysis in showing that the three-cluster grouping should not be interpreted as the statistically optimal partition solely on the basis of internal cluster-separation criteria.

For the multidimensional K=3 solution, Bishnupur, Imphal East, Imphal West, and Thoubal formed one cluster; Churachandpur constituted a separate cluster; and Chandel, Senapati, Tamenglong, and Ukhrul formed the third cluster. Comparison of this indicator-profile-based partition with the primary K=3 grouping derived from the composite index yielded an Adjusted Rand Index (ARI) of 0.2041, indicating limited agreement between the two groupings. This suggests that districts occupying similar positions on the one-dimensional composite index do not necessarily exhibit similar patterns across all eleven underlying maternal and child health performance indicators.

Nevertheless, once K=3 was prespecified, the multidimensional partition showed high computational stability. As reported in Table 10, the K=3 solution showed complete agreement with Ward.D2 hierarchical clustering (ARI = 1.000), and the random-initialization sensitivity analysis produced a mean ARI of 1.000 with 100% perfect agreement across 100 seeds. The same level of algorithmic stability was observed for the K=2 and K=4 solutions. These findings indicate that the identified partitions were reproducible for each prespecified value of K, but such stability should be distinguished from evidence concerning the optimal number of clusters.

Overall, the multidimensional sensitivity analysis provides an important qualification to the primary score-based grouping. The relatively low agreement between the composite-index and indicator-profile K=3 partitions indicates that the primary clusters are better interpreted as exploratory strata of overall composite performance rather than as homogeneous multidimensional maternal and child health performance typologies. Accordingly, the composite-index grouping should be considered together with the underlying district-specific indicator profiles when interpreting inter-district differences.

## 4. Discussion

The present study provides a multidimensional assessment of maternal and child health performance across nine districts of Manipur by integrating eleven indicators through the CRITIC–TOPSIS framework. The findings reveal considerable inter-district heterogeneity in maternal healthcare utilization, postnatal care, immunization, breastfeeding practices, and child nutritional outcomes. Rather than indicating a simple geographical dichotomy, the results show a differentiated pattern, with Imphal West recording the highest composite performance, five districts occupying intermediate positions, and Senapati, Chandel, and Ukhrul exhibiting comparatively lower performance. This variation is consistent with evidence of substantial subnational disparities in health outcomes and healthcare provision across India [18,20,22].

A prominent feature of the findings is the strong co-variation among indicators representing successive components of the maternal–newborn–child continuum of care. Maternal and newborn postnatal care showed the strongest positive association, while institutional delivery was also strongly associated with both maternal and newborn postnatal care. ANC with four or more visits was positively associated with first-trimester ANC, institutional delivery, basic vaccination, and postnatal care. These patterns suggest that districts with relatively favorable coverage of one component of maternal healthcare also tended to perform favorably on other components, consistent with Indian evidence emphasizing continuity across antenatal, delivery, postnatal, and child healthcare services [13,14]. These associations, however, represent district-level co-variation and should not be interpreted as individual-level relationships or evidence of causality.

Child nutritional outcomes represented an additional and partly distinct dimension of district-level health performance. Stunting varied appreciably across districts and was inversely associated with maternal postnatal care, institutional delivery, and newborn postnatal care, while first-trimester ANC was inversely associated with wasting. Relationships among stunting, wasting, and underweight were not uniformly strong, indicating that these indicators capture different dimensions of child nutritional status. This finding is consistent with evidence of spatial heterogeneity in child growth failure across India and its association with household and contextual characteristics [15]. Such contextual factors were not directly modelled in the present study and should therefore not be interpreted as mechanisms explaining the observed district-level differences.

The CRITIC-derived weights illustrate the importance of considering both variability and redundancy when constructing a multidimensional index. Breastfeeding within one hour received the largest weight (0.1904), followed by wasting (0.1269), exclusive breastfeeding (0.1039), stunting (0.0994), and underweight (0.0931). Under CRITIC, weights reflect the contrast intensity of an indicator and its degree of conflict or redundancy with other criteria [32]. Accordingly, the relatively high weight assigned to early initiation of breastfeeding reflects its distinctive statistical information contribution within the indicator system and should not be interpreted as evidence of greater clinical importance. Conversely, the comparatively lower weights assigned to several maternal healthcare indicators are consistent with their strong intercorrelations and greater information redundancy. This distinction is important because statistical weighting and substantive or clinical importance represent different concepts [30,32].

The TOPSIS results further demonstrate differences in relative district performance. Imphal West recorded the highest composite index (0.8674), followed by Bishnupur (0.5851) and Imphal East (0.5564), whereas Ukhrul recorded the lowest value (0.3499). Importantly, some adjacent ranks corresponded to very similar composite scores; for example, Churachandpur (0.5173) and Thoubal (0.5167) differed only marginally. District rankings should therefore be interpreted together with the magnitude of the underlying composite scores rather than as evidence of substantial differences between adjacent ranks. The coefficient of variation (29.17%) indicates appreciable relative dispersion, whereas the Gini coefficient (0.1411), Theil T (0.0350), and Theil L (0.0333) suggest comparatively limited inequality across the overall distribution. Together, these measures indicate one distinctly high-performing district alongside a broader continuum of intermediate and comparatively lower-performing districts. Such use of composite indicators to summarize multiple dimensions is consistent with multicriteria approaches used in regional and developmental assessment [30,36,37,38,39].

The sensitivity analyses provide additional support for the robustness of the broad district-ranking pattern. Replacing the CRITIC-derived weights with equal weights produced strong agreement with the baseline ranking (Spearman’s ρ=0.9500), with a maximum rank displacement of only one position. Similarly, the leave-one-district-out analysis produced a mean Spearman rank correlation of 0.9841 across the nine omission scenarios, with correlations ranging from 0.9524 to 1.000 and a maximum displacement of one position. These findings indicate that the broad ranking structure was not materially dependent on either the CRITIC weighting specification or the inclusion of any single district. Nevertheless, minor changes among closely ranked districts reinforce the need to interpret small ordinal differences cautiously.

The exploratory clustering analysis provides an additional descriptive perspective on the distribution of composite performance. The retained K = 3 solution grouped Imphal West separately, placed Bishnupur, Imphal East, Churachandpur, Thoubal, and Tamenglong together, and grouped Senapati, Chandel, and Ukhrul at comparatively lower levels of composite performance. The inclusion of Churachandpur and Tamenglong in the intermediate group suggests that the observed pattern cannot be explained solely by a simple valley-hill geographical division. Although geographical accessibility, healthcare infrastructure, transportation, and socioeconomic conditions may contribute to health disparities in Manipur and other Indian settings [18,19,20,22], these factors were not directly modelled and should be regarded as contextual influences rather than causal determinants established by the present study.

The K = 3 solution should nevertheless be interpreted cautiously. The K = 2 solution produced the highest average silhouette width (0.6528), compared with 0.5602 for K = 3 and 0.5047 for K = 4, but primarily separated Imphal West from the remaining eight districts. The K = 3 solution was therefore retained as an exploratory grouping because it provided greater differentiation among the remaining districts while maintaining a positive average silhouette width. Its reproducibility was supported by complete agreement with Ward.D2 hierarchical clustering (ARI = 1.000) and stability across random initializations. However, the multidimensional indicator-profile sensitivity analysis showed limited agreement with the composite-index-based K = 3 partition (ARI = 0.2041). The retained clusters should therefore be interpreted as exploratory strata of overall composite performance rather than homogeneous multidimensional health typologies. This distinction reinforces the importance of interpreting composite scores and cluster groupings together with the underlying district-specific indicator profiles [30,31].

Taken together, the findings demonstrate the value of geographically disaggregated, multidimensional assessment for identifying relative differences in maternal and child health performance that may be obscured by state-level summaries. NFHS-5 provides district-level information across maternal healthcare, child healthcare, breastfeeding, and nutritional indicators [16], while previous studies have documented substantial spatial variation in health outcomes and healthcare provision across India [18,20,22]. The present framework complements such evidence by integrating multiple dimensions into a transparent district-level assessment. However, the resulting scores, rankings, and exploratory groupings are relative to the nine districts and eleven indicators included in the analysis and should not be interpreted as absolute clinical thresholds, definitive measures of health-system performance, or evidence of causal relationships.

## 5. Limitations

This study has several limitations. The analysis is based on only nine districts and cross-sectional NFHS-5 (2019–2021) data; therefore, the findings are descriptive and do not support causal or individual-level inference. The small number of districts may also affect the stability of inter-indicator correlations and cluster-validation measures. The eleven indicators were constrained by the availability of comparable district-level data, and the resulting composite scores and rankings remain specification-dependent on the selected indicators, normalization, weighting structure, and TOPSIS formulation, although the sensitivity analyses indicated substantial ranking stability. Further, the NFHS-5 data represent conditions during 2019–2021 and may not reflect subsequent changes in district-level maternal and child health performance. Finally, the limited agreement between composite-index and multidimensional clustering (ARI = 0.2041) indicates that the retained clusters should be interpreted as exploratory groupings rather than definitive health typologies.

## 6. Policy Implications

The findings support a differentiated district-level approach to maternal and child health planning in Manipur rather than relying solely on state-level averages or broad geographical groupings. Policy interpretation should primarily focus on the eleven indicators assessed in this study. Districts with comparatively lower composite performance, particularly Senapati, Chandel, and Ukhrul, warrant closer examination of their indicator-specific profiles across antenatal care, institutional delivery, maternal and newborn postnatal care, childhood immunization, breastfeeding practices, and child nutritional outcomes. Such profiles can help identify the specific dimensions contributing to relatively lower performance and provide a more informative basis for district-level prioritization than composite scores or cluster membership alone.

For districts with intermediate composite performance, policy attention should similarly be guided by the particular indicators showing relatively weaker performance. Given the established importance of continuity across maternal and child healthcare services [13,14], the findings may support closer assessment of gaps across antenatal, delivery, postnatal, immunization, breastfeeding, and nutritional dimensions. Imphal West, as the highest-performing district in the composite assessment, may provide a useful internal benchmark; however, its practices and contextual conditions should be independently examined before considering their applicability to other districts. Periodic monitoring of both composite performance and individual indicators may also help identify changing district-level patterns over time.

Broader health-system measures related to healthcare accessibility, infrastructure, referral mechanisms, outreach, transportation, and digital health services may be relevant in geographically challenging settings. However, these factors were not directly measured or modelled in the present study and therefore cannot be identified as determinants of the observed district-level differences. They should instead be regarded as contextual considerations informed by previous literature [16,18,19,20]. Further research incorporating healthcare infrastructure, geographical accessibility, socioeconomic conditions, and health-system capacity is required to examine their contribution to the observed disparities and to evaluate the appropriateness of specific interventions.

Overall, the Composite Maternal and Child Health Performance Index should be regarded primarily as a screening and prioritization tool for relative district-level assessment, rather than as sufficient evidence for prescribing interventions or determining resource allocation. Composite scores and exploratory cluster groupings should be interpreted alongside the underlying indicator profiles, local contextual evidence, and programme-level information before district-specific actions are considered.

## 7. Conclusions

This study demonstrates notable inter-district variation in maternal and child health performance across Manipur using the CRITIC–TOPSIS framework. Imphal West recorded the highest composite score, while Ukhrul recorded the lowest. The resulting scores and rankings represent relative multidimensional performance across the nine districts and eleven indicators included in the analysis and should not be interpreted as absolute or definitive measures of overall maternal and child health. The K = 3 solution provides an exploratory grouping of districts according to their composite performance rather than a validated developmental or clinical classification. Given the small number of districts and the cross-sectional nature of the NFHS-5 data, the findings should be interpreted as descriptive and exploratory rather than causal. Nevertheless, the framework provides a reproducible approach for district-level screening and prioritization when interpreted alongside underlying indicator profiles, local contextual factors, and programme-level evidence. Future research may extend the framework to a larger number of geographical units and multiple survey rounds and incorporate healthcare infrastructure, geographical accessibility, socioeconomic conditions, and other contextual determinants to examine the drivers and temporal evolution of maternal and child health disparities.

## Figures and Tables

**Figure 1:**
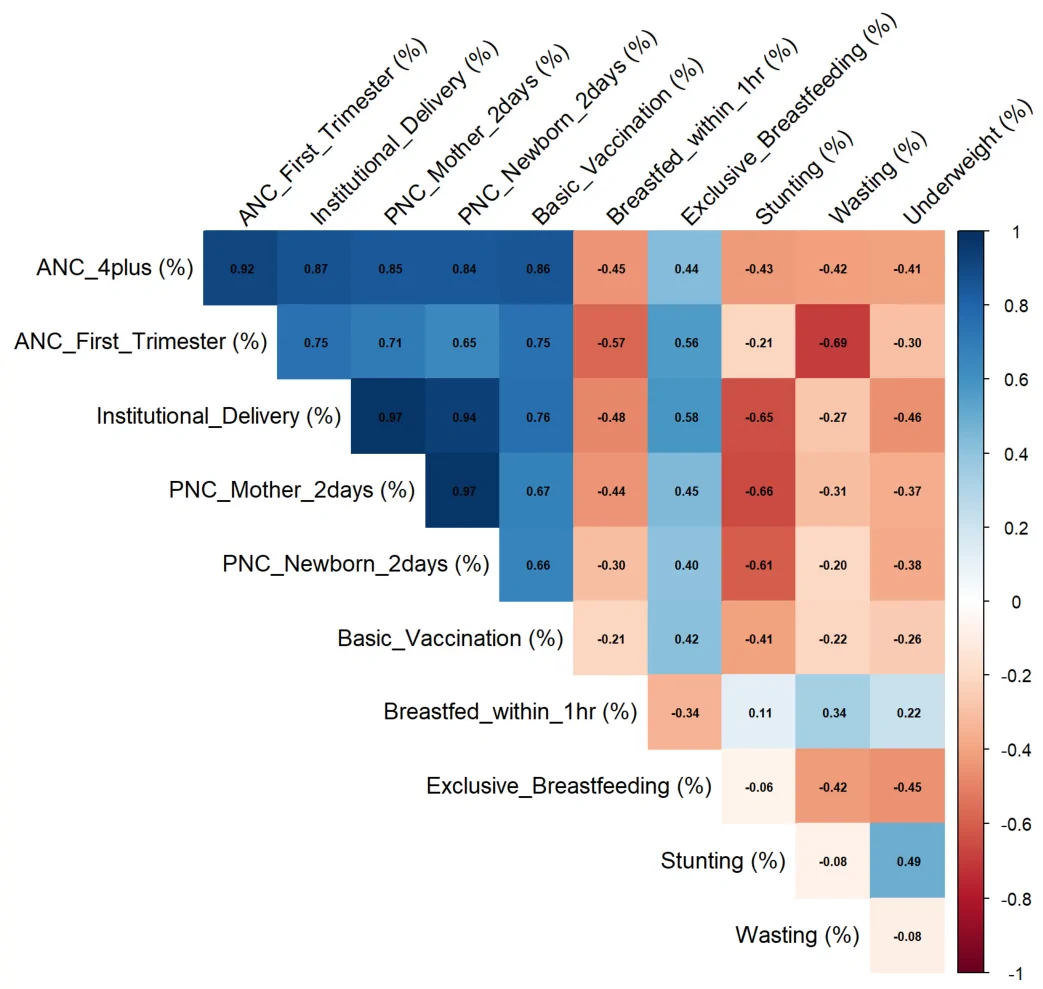
Heatmap of Pearson Correlations among Maternal and Child Health Performance Indicators across Districts of Manipur. Figure note: The heatmap represents Pearson correlation coefficients calculated from the nine district-level observations. The coefficients describe the direction and magnitude of linear associations and should not be interpreted as evidence of statistical significance or causality.

**Figure 2:**
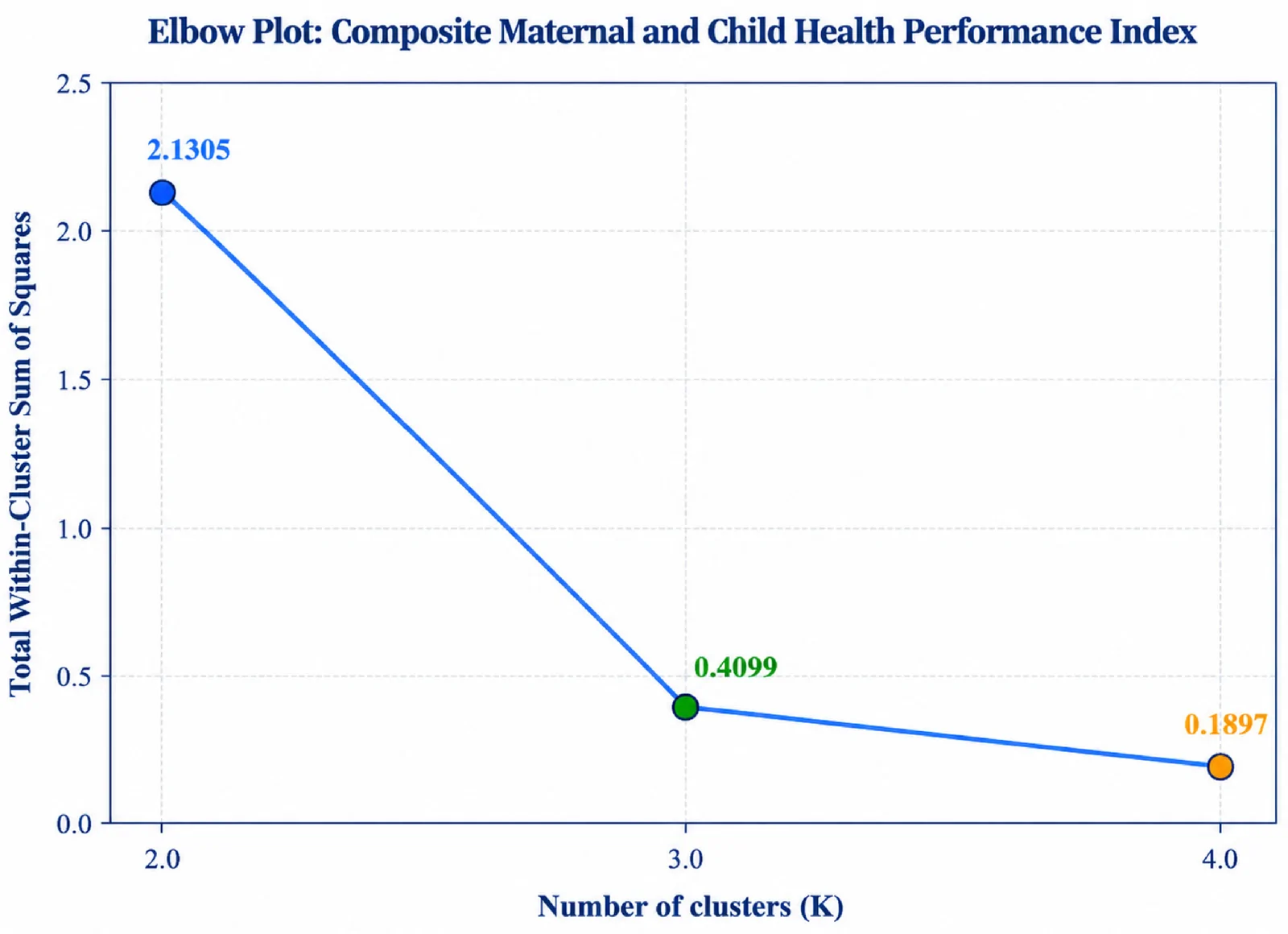
Elbow Plot for K-Means Clustering of the Composite Maternal and Child Health Performance Index.

**Figure 3:**
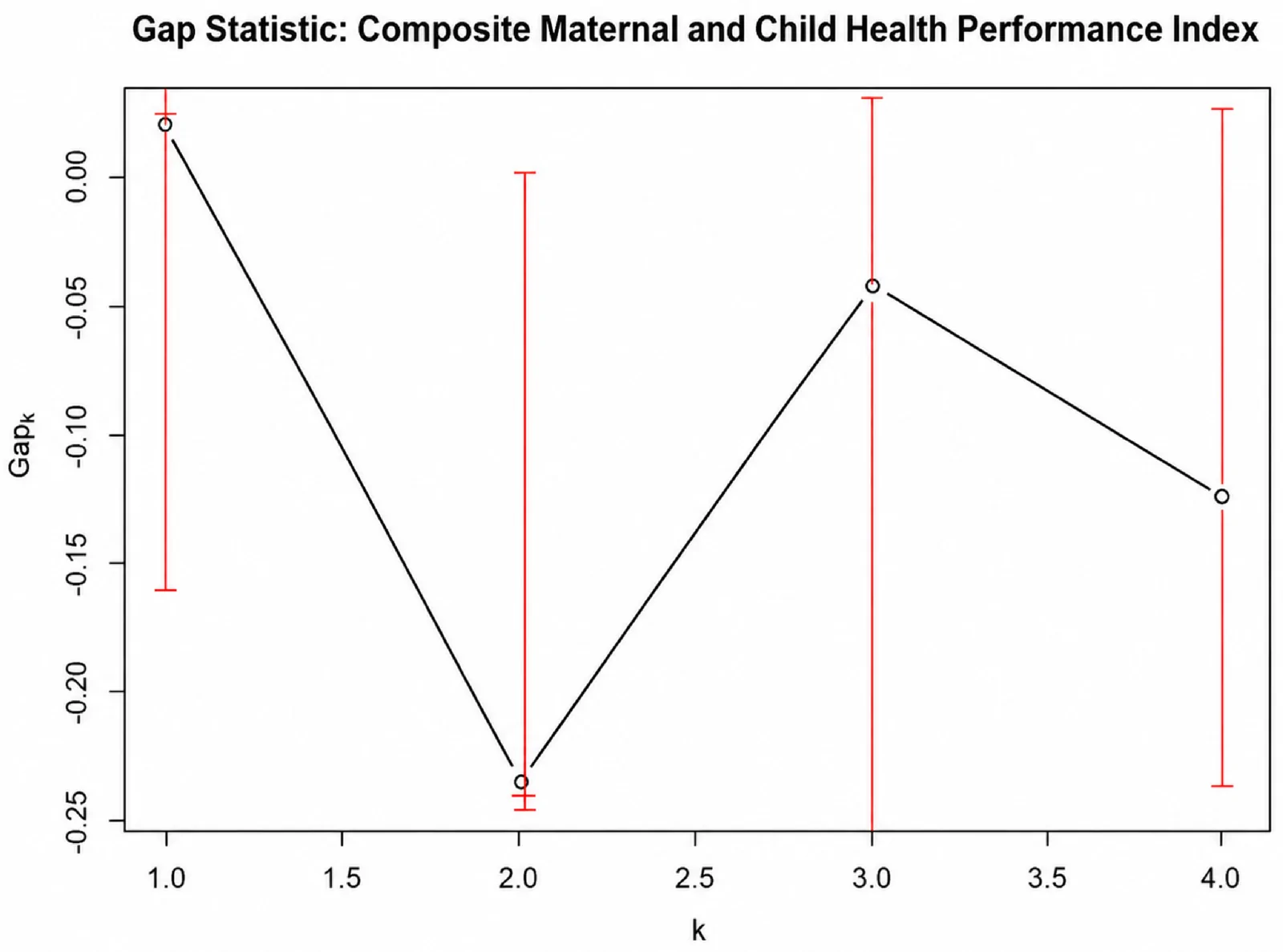
Gap Statistic for Alternative Cluster Solutions of the Composite Maternal and Child Health Performance Index.

**Figure 4:**
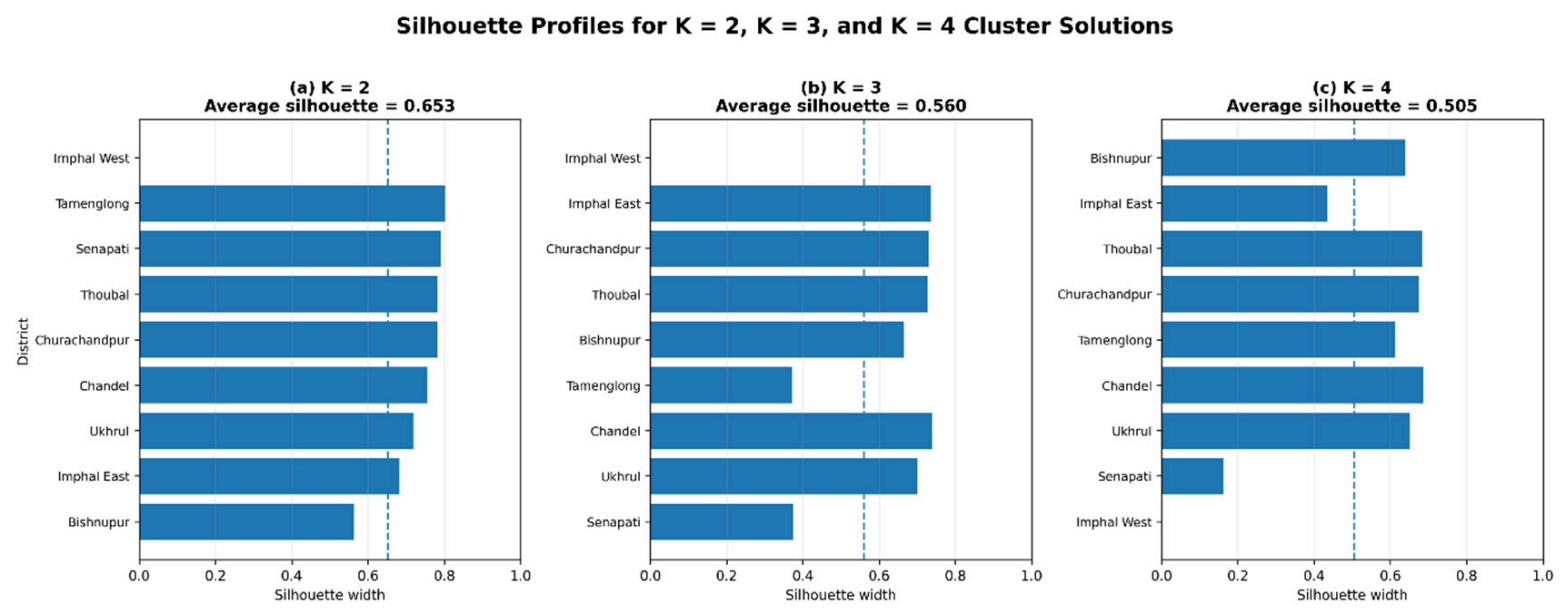
Silhouette Profiles for K=2, K=3, and K=4 Cluster Solutions. Note: Average silhouette widths were 0.6528, 0.5602, and 0.5047 for K=2, K=3, and K=4, respectively. A silhouette width of zero for the singleton cluster reflects the absence of within-cluster neighbors and should not be interpreted as negative assignment.

**Figure 5:**
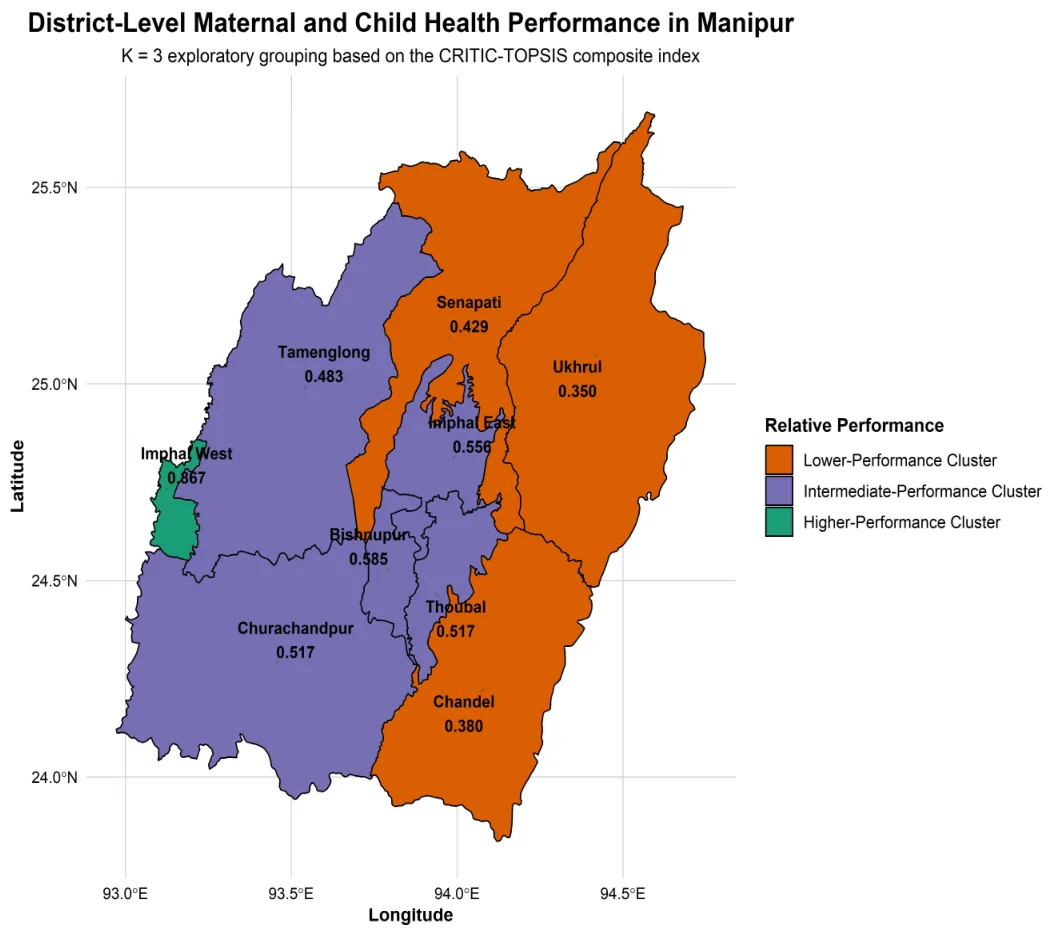
Spatial distribution of the composite-index-based maternal and child health performance groups across the nine districts of Manipur using the 2011 administrative district framework [41]. Note: District boundaries correspond to the Census of India 2011 administrative district framework used for the nine district units included in the analysis. Imphal East and Imphal West are separate adjoining administrative districts under this framework.

**Figure 6:**
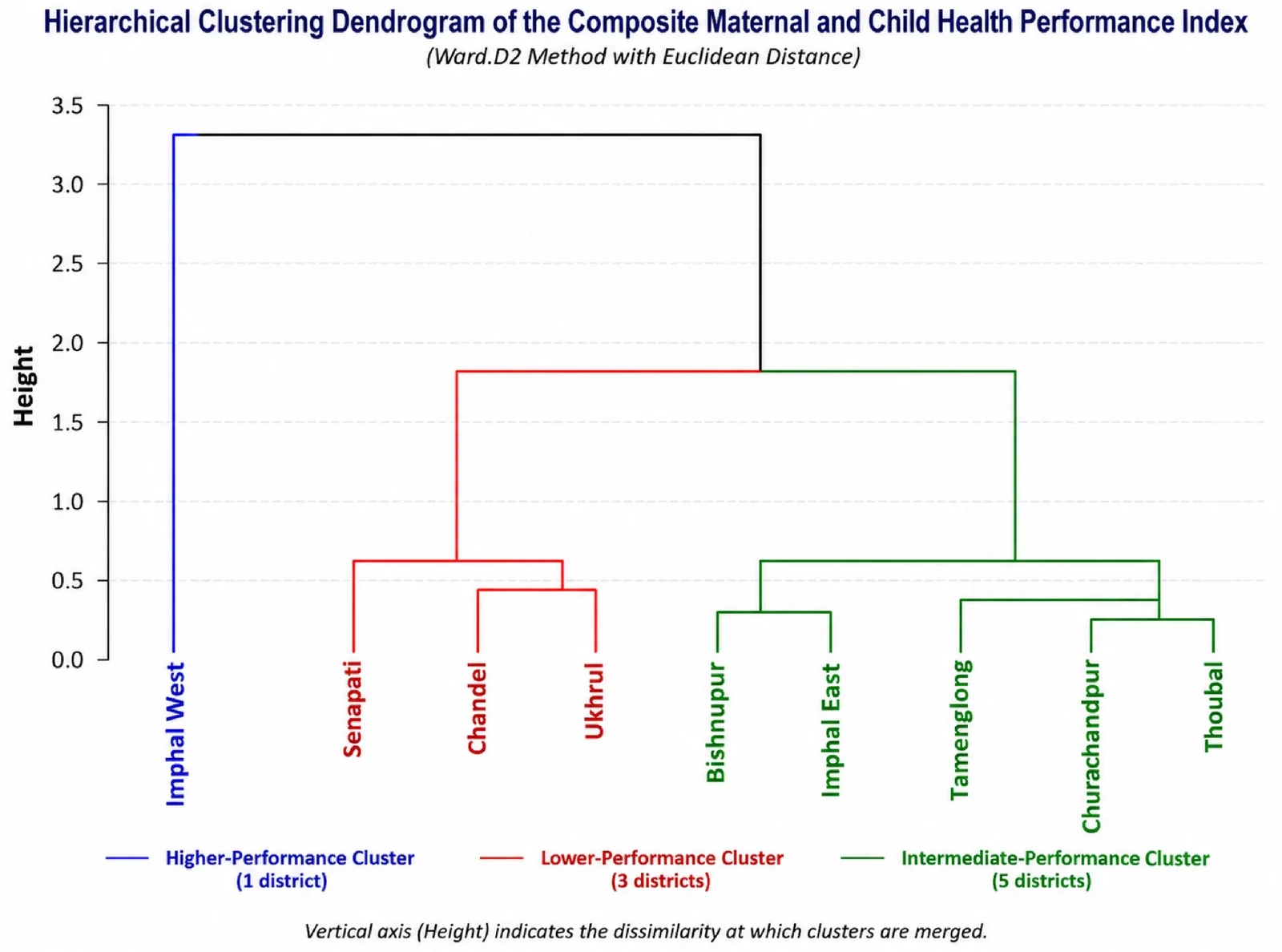
Ward.D2 Hierarchical Clustering Dendrogram of the Composite Maternal and Child Health Performance Index.

**Figure 7:**
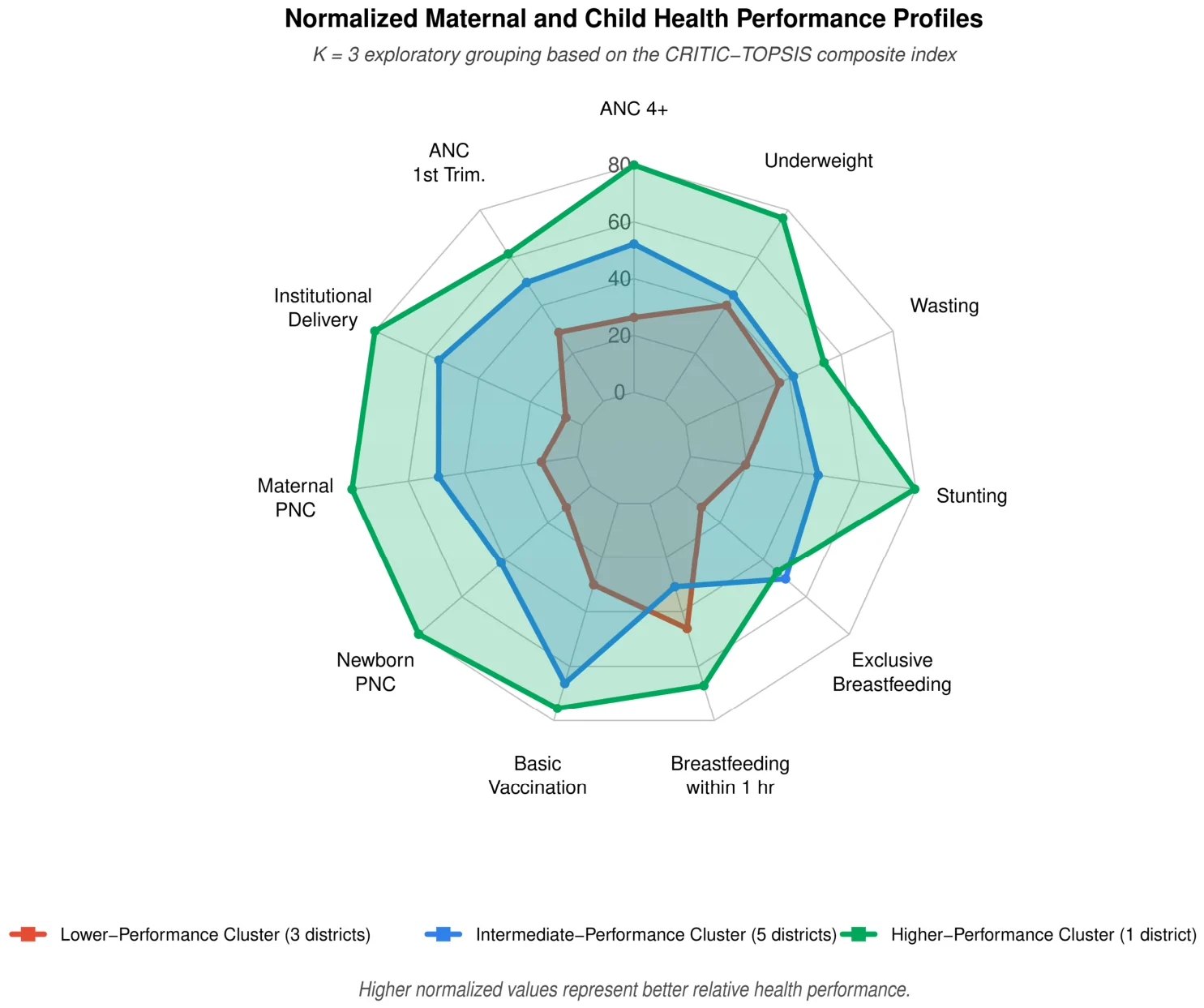
Normalized Maternal and Child Health Indicator Profiles across the Composite-Performance Clusters. Note: Radar values are normalized cluster profiles and should not be interpreted as raw percentages. Stunting, wasting, and underweight were direction-adjusted so that higher normalized values represent more favorable nutritional outcomes.

**Table 1:** Classification and Direction of Maternal and Child Health Performance Indicators.

Indicator	Performance Direction	Criteria
ANC 4+ visits	Higher is Better	Benefit Criteria
ANC registration in 1st trimester	Higher is Better	Benefit Criteria
Institutional delivery	Higher is Better	Benefit Criteria
PNC for mother within 2 days	Higher is Better	Benefit Criteria
PNC for newborn within 2 days	Higher is Better	Benefit Criteria
Basic Vaccination	Higher is Better	Benefit Criteria
Breastfeeding initiation within 1 h	Higher is Better	Benefit Criteria
Exclusive breastfeeding	Higher is Better	Benefit Criteria
Stunting	Lower is Better	Cost Criteria
Wasting	Lower is Better	Cost Criteria
Underweight	Lower is Better	Cost Criteria

**Table 2:** Descriptive Statistics of Maternal and Child Health Performance Indicators across Districts of Manipur.

Indicator	N	Mean	SD	Median	Min	Max	Range	Skewness	Kurtosis	SE	CV (%)
ANC 4+	9	70.6444	17.8839	66.6	38.8	93.4	54.6	−0.2441	−1.3061	5.9613	25.3153
ANC First Trimester	9	76.0889	9.6937	77.6	58.3	90.6	32.3	−0.2838	−1.0375	3.2312	12.7400
Institutional Delivery	9	71.2444	20.6618	72.9	44.6	95.7	51.1	−0.1003	−1.9409	6.8873	29.0013
PNC Mother within 2 days	9	64.2889	18.9445	58.2	37.0	90.8	53.8	0.0405	−1.7246	6.3148	29.4677
PNC Newborn within 2 days	9	60.9889	18.1910	56.8	36.0	91.7	55.7	0.3002	−1.4182	6.0637	29.8268
Basic Vaccination	9	64.8111	11.4374	66.6	39.4	76.2	36.8	−1.0461	−0.0354	3.8125	17.6472
Breastfed within 1 h	9	55.2556	8.6946	57.2	42.6	67.9	25.3	−0.0469	−1.6902	2.8982	15.7352
Exclusive Breastfeeding	9	69.8444	5.6292	68.6	63.1	77.7	14.6	0.1608	−1.7776	1.8764	8.0596
Stunting	9	24.6222	6.6919	27.1	15.5	34.0	18.5	−0.1982	−1.6067	2.2306	27.1784
Wasting	9	9.9444	1.6264	9.6	7.9	12.1	4.2	0.0684	−1.8693	0.5421	16.3552
Underweight	9	13.7667	2.9925	14.4	9.8	19.3	9.5	0.2729	−1.0698	0.9975	21.7372

**Table 3:** Pearson Correlation Matrix of Maternal and Child Health Performance Indicators across Districts of Manipur.

Indicator	ANC4	ANC1	ID	PNC-M	PNC-N	BV	BF1h	EBF	ST	WA	UW
**ANC4**	1.000	0.917	0.870	0.849	0.843	0.855	−0.448	0.438	−0.427	−0.416	−0.408
**ANC1**	0.917	1.000	0.748	0.705	0.648	0.750	−0.574	0.565	−0.211	−0.691	−0.299
**ID**	0.870	0.748	1.000	0.971	0.936	0.758	−0.482	0.584	−0.647	−0.274	−0.459
**PNC-M**	0.849	0.705	0.971	1.000	0.974	0.671	−0.444	0.446	−0.657	−0.305	−0.368
**PNC-N**	0.843	0.648	0.936	0.974	1.000	0.662	−0.301	0.401	−0.605	−0.201	−0.378
**BV**	0.855	0.750	0.758	0.671	0.662	1.000	−0.214	0.417	−0.408	−0.218	−0.258
**BF1h**	−0.448	−0.574	−0.482	−0.444	−0.301	−0.214	1.000	−0.344	0.114	0.339	0.224
**EBF**	0.438	0.565	0.584	0.446	0.401	0.417	-0.344	1.000	−0.060	−0.422	−0.452
**ST**	−0.427	−0.211	−0.647	−0.657	−0.605	−0.408	0.114	−0.060	1.000	−0.079	0.491
**WA**	−0.416	−0.691	−0.274	−0.305	−0.201	−0.218	0.339	−0.422	−0.079	1.000	−0.082
**UW**	−0.408	−0.299	−0.459	−0.368	−0.378	−0.258	0.224	−0.452	0.491	−0.082	1.000

***Note:*** *ANC4 = ANC 4+ visits; ANC1 = ANC during first trimester; ID = Institutional Delivery; PNC-M = Maternal PNC within two days; PNC-N = Newborn PNC within two days; BV = Basic Vaccination; BF1h = Breastfed within one hour; EBF = Exclusive Breastfeeding; ST = Stunting; WA = Wasting; UW = Underweight. Correlations are based on nine district-level observations.*

**Table 4:** CRITIC-Derived Weights of Maternal and Child Health Performance Indicators.

Indicator	Normalized SD	Conflict	Information Content	CRITIC Weight
ANC 4+	0.327543	4.424319	1.449156	0.059553
ANC First Trimester	0.300116	5.039179	1.512337	0.062149
Institutional Delivery	0.404341	4.237298	1.713312	0.070408
PNC Mother within 2 days	0.352128	4.496535	1.583356	0.065068
PNC Newborn within 2 days	0.326589	4.652927	1.519597	0.062448
Basic Vaccination	0.310798	5.218202	1.621808	0.066648
Breastfed within 1 h	0.343658	13.484723	4.634137	0.190439
Exclusive Breastfeeding	0.385561	6.558039	2.528524	0.103909
Stunting	0.361726	6.686711	2.418757	0.099398
Wasting	0.387245	7.971579	3.086958	0.126858
Underweight	0.314999	7.193777	2.266033	0.093122

**Table 5:** TOPSIS-Based Composite Maternal and Child Health Performance Index and District Rankings.

Rank	District	$D_{i}^{+}$	$D_{i}^{-}$	Composite Index
**1**	Imphal West	0.008410	0.055009	0.867390
**2**	Bishnupur	0.031194	0.043986	0.585077
**3**	Imphal East	0.031948	0.040076	0.556428
**4**	Churachandpur	0.032219	0.034531	0.517312
**5**	Thoubal	0.034311	0.036680	0.516681
**6**	Tamenglong	0.037177	0.034746	0.483098
**7**	Senapati	0.039108	0.029390	0.429067
**8**	Chandel	0.041175	0.025216	0.379810
**9**	Ukhrul	0.044253	0.023816	0.349877

**Table 6:** Disparity Measures for the District-Level Composite Maternal and Child Health Performance Index.

Measure	Value
Mean	0.520527
Standard Deviation	0.151857
Minimum	0.349877
Maximum	0.867390
Absolute Range	0.517513
Coefficient of Variation (%)	29.1737
IQR	0.127362
Gini coefficient	0.141110
Theil T	0.034954
Theil L	0.033294

**Table 7:** Sensitivity and Robustness of the CRITIC–TOPSIS Composite Index.

Sensitivity Analysis	Ranking Agreement with Baseline	Range of Spearman’s ρ	Maximum Rank Displacement
Equal-weight TOPSIS	ρ = 0.9500	—	1
Leave-one-district-out CRITIC–TOPSIS	Mean ρ = 0.9841	0.9524–1.000	1

*Note: For the leave-one-district-out analysis, Spearman’s rank correlation was calculated between each recalculated eight-district ranking and the corresponding baseline ranking restricted to the same eight districts. The reported value of 0.9841 represents the mean across the nine district-omission scenarios.*

**Table 8:** Comparison of Cluster-Validation Diagnostics for K=2, K=3 and K=4.

K	WSS	WSS Reduction from Previous K	Average Silhouette	Negative Silhouette (%)	Ward.D2 ARI	Mean Seed ARI	Perfect Seed Agreement (%)	Cluster Size Range
2	2.1305	-	0.6528	0	1.000	1.000	100	1–8
3	0.4099	80.76%	0.5602	0	1.000	1.000	100	1–5
4	0.1897	53.73%	0.5047	0	1.000	1.000	100	1–3

**Table 9:** District Grouping and Composite Index Profile under the K = 3 Solution.

Cluster Category	District	Composite Index
Higher-Performance	Imphal West	0.867390
Intermediate-Performance	Bishnupur	0.585077
	Imphal East	0.556428
	Churachandpur	0.517312
	Thoubal	0.516681
	Tamenglong	0.483098
Lower-Performance	Senapati	0.429067
	Chandel	0.379810
	Ukhrul	0.349877

**Table 10:** Sensitivity Analysis of Multidimensional Clustering Based on the 11 Indicator Profiles.

K	WSS	Average Silhouette Width	Minimum Silhouette Width	Ward.D2 ARI	Mean Seed ARI	Perfect Seed Agreement (%)
2	45.3388	**0.3184**	0.0801	1.000	1.000	100
3	35.5590	0.1651	0.0000	1.000	1.000	100
4	26.8155	0.1407	0.0000	1.000	1.000	100

***Note:*** *WSS = within-cluster sum of squares; ARI = Adjusted Rand Index. For *K=3*, the ARI comparing the multidimensional indicator-profile partition with the composite-index-based partition was 0.2041. Seed stability was assessed across 100 random seeds.*

## Data Availability

The data used in this study were obtained from the National Family Health Survey-5 (NFHS-5), 2019–2021, conducted by the International Institute for Population Sciences under the Ministry of Health and Family Welfare, Government of India. The data are publicly available through the NFHS data portal at: https://www.nfhsiips.in/nfhsuser/index.php (accessed on 20 January 2026).

## Supplementary Materials

The following supporting information can be downloaded at: https://www.mdpi.com/article/10.3390/healthcare14193143/s1. The following supporting information is available with this article: Supplementary File S1, containing the complete district-level dataset for the eleven maternal and child health indicators included in the analysis, along with the CRITIC weights, TOPSIS results, disparity measures, and outputs from the sensitivity and clustering analyses; and Supplementary File S2, containing the R code used to reproduce the CRITIC weighting, TOPSIS composite index, disparity measures, K-means and hierarchical clustering analyses, and sensitivity and robustness analyses.
